# The Role of Selective Protein Degradation in the Regulation of Iron and Sulfur Homeostasis in Plants

**DOI:** 10.3390/ijms21082771

**Published:** 2020-04-16

**Authors:** Anna Wawrzyńska, Agnieszka Sirko

**Affiliations:** Institute of Biochemistry and Biophysics Polish Academy of Sciences, 02-106 Warsaw, Poland; asirko@ibb.waw.pl

**Keywords:** autophagy, iron metabolism, proteasome, protein degradation, sulfur metabolism, ubiquitin

## Abstract

Plants are able to synthesize all essential metabolites from minerals, water, and light to complete their life cycle. This plasticity comes at a high energy cost, and therefore, plants need to tightly allocate resources in order to control their economy. Being sessile, plants can only adapt to fluctuating environmental conditions, relying on quality control mechanisms. The remodeling of cellular components plays a crucial role, not only in response to stress, but also in normal plant development. Dynamic protein turnover is ensured through regulated protein synthesis and degradation processes. To effectively target a wide range of proteins for degradation, plants utilize two mechanistically-distinct, but largely complementary systems: the 26S proteasome and the autophagy. As both proteasomal- and autophagy-mediated protein degradation use ubiquitin as an essential signal of substrate recognition, they share ubiquitin conjugation machinery and downstream ubiquitin recognition modules. Recent progress has been made in understanding the cellular homeostasis of iron and sulfur metabolisms individually, and growing evidence indicates that complex crosstalk exists between iron and sulfur networks. In this review, we highlight the latest publications elucidating the role of selective protein degradation in the control of iron and sulfur metabolism during plant development, as well as environmental stresses.

## 1. Introduction

Because plants are sessile and often face biotic and abiotic stresses, including fluctuating availability of nutrients, drought, and diseases, their ability to thrive in a dynamic environment is a biochemical feat. Nonetheless, this plasticity comes at a high price. Plants are continuously pressured to recognize and allocate adequate resources to metabolic processes such as growth, development, and the assimilation of nutrients. They also need to determine when to slow or even stop development and growth if resources are low (i.e, nutrient restriction) and if they need to divert resources to support other essential processes, such as defense [1,2]. Plants have developed mechanisms to sense the availability of nutrients and, consequently, regulate their growth and development. They are also able to store excess nutrients and diminish their uptake if storage capacity is exceeded, to prevent the accumulation of nutrients to toxic levels (for reviews, see [3,4]). This is of particular importance for reactive elements such as iron and copper, which are required in relatively small amounts (micromolar range), but can become toxic at a relatively low level (submillimolar ranges). It has become clear in recent years that there is active crosstalk between networks regulating the uptake and use of nutrients [5,6,7].

**Figure 1:**
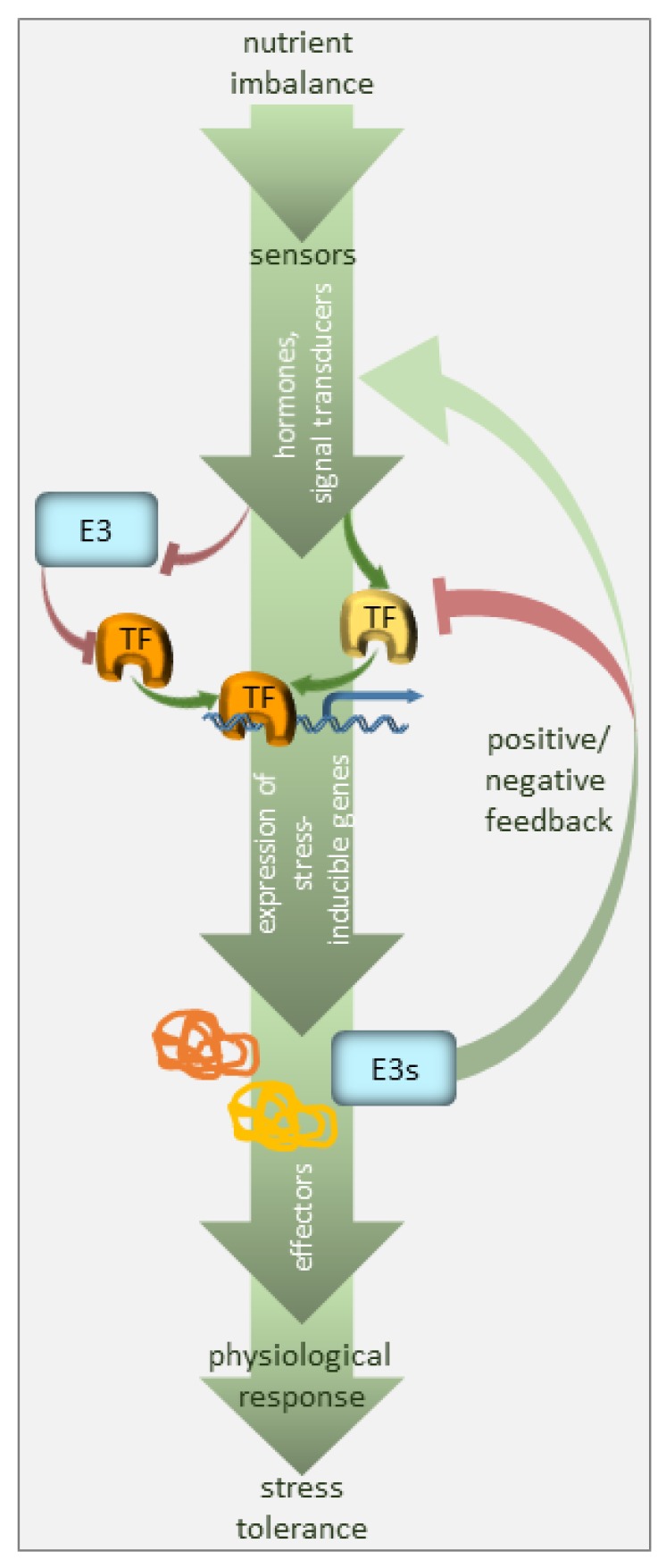
Regulation of nutrient deficiency stress by ubiquitination. The plant perceives nutrient deficiency via sensors (not defined in the model) and the signal is transduced via phytohormones, secondary messengers, and transcriptional regulators. The transcription factors (TF), many of which are stress-regulated, facilitate the expression of stress-inducible genes. E3 ubiquitin ligases target the components of the signaling pathway, i.e., mainly stress-responsive TFs, promoting their degradation, and thus, suppressing the pathway in the absence of the signal. E3 ubiquitin ligases may also serve as a feedback mechanism to enhance or silence the stress signal.

Homeostasis is a term that has been used for nearly a century; it explains processes used by systems to sustain survival conditions [8]. Optimal growth and development are only achieved when nutrients are present at certain concentrations, i.e., when the demand meets the supply. Below such levels, the demand for a nutrient exceeds the supply, so plant growth is ultimately limited by a nutritional deficiency. Conversely, an excess of any nutrient is toxic to plants and also negatively affects plant growth and development. Plants can therefore experience transitions between deficiency or toxicity zones due to changes in the availability of nutrients and growth requirements, but they can return to homeostasis so long as the response is adequate and sufficient. Such adjustments may require time, but they are crucial for plant adaptation. A significant part of this adaptation relies on selective protein degradation (Figure 1).

**Figure 2:**
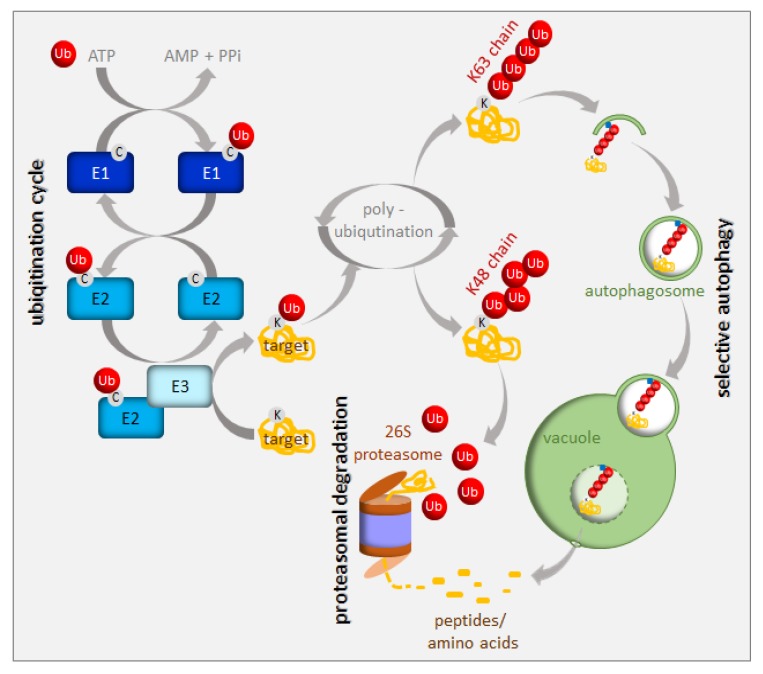
Ubiquitination and degradation of ubiquitinated proteins. Ubiquitin (Ub) is activated and conjugated to the target protein by a series of E1 (ubiquitin-activating enzyme), E2 (ubiquitin-conjugating enzyme), and E3 (ubiquitin ligase) activities. Additional ubiquitin molecules can be added to the monoubiquitinated target (polyubiquitination). The polyubiquitinated proteins are recruited either to the 26S proteasome or to the autophagy pathway depending on their polyubiquitin chain structure. See the text for more details.

Protein turnover has been estimated to account for up to one-third of the total energy cost in fast-growing cells [9]. Consequently, an immediate cell reaction to various stresses, including nutrient starvation, is translation arrest. This thereby frees energy and resources for stress responses. A small, highly-conserved 76-amino-acid protein called ubiquitin is a tag marking soluble proteins for degradation. The process of ubiquitination requires the sequential action of three enzymes, i.e., E1, E2, and E3 [10] (Figure 2). In the first step of this enzymatic cascade, ubiquitin is bound to a cysteine residue of the activating enzyme (E1) through its C-terminal glycine, forming a thioester bond. Subsequently, activated ubiquitin is transferred to the conjugating enzyme (E2), also via a thioester bond. A member of the ubiquitin ligase family (E3) then recognizes the substrate for ubiquitination and catalyzes the transfer of ubiquitin from the E2 enzyme to the substrate protein. The cascade of ubiquitination has to be highly selective, and its accuracy depends on a sufficient number of E3 enzymes; hence, E3 ubiquitin ligases represent the largest protein family in plants. Plants use two mechanistically distinct but largely complementary systems to selectively target a wide range of proteins for degradation: the ubiquitin-proteasome system (UPS) and selective autophagy. The concerted action of the ubiquitination machinery in both systems ensures the targeted and tightly regulated degradation of cellular proteins. These degradation mechanisms can be perceived as a housecleaning process, where old, damaged, or no-longer-needed proteins, polypeptides, organelles, lipids, and other cellular components are recycled and reused. However, they are also of crucial importance when growth conditions get tough and metabolism remodeling is urgently needed. The UPS and autophagy are differentially regulated and usually degrade different protein sets. Proteins marked by K48-linked polyubiquitin are commonly believed to have a high affinity for the 26S proteasome [11], whereas those with a K63-polyubiquitin or monoubiquitin tag are preferred substrates for autophagy clearance [12,13,14] (Figure 2). The two systems of protein degradation are not fully independent; moreover, their functions must be coordinated, since both pathways are responsible for homeostasis of cellular proteins.

The UPS is responsible for the rapid destruction of a wide range of intracellular regulators (usually short-lived proteins) via the 26S proteasome. Therefore, the UPS plays an important role in cell signaling, hormone biogenesis and responses, chromatin structure regulation and transcription, and even pathogenic infections (for a review see [15]). The 26S proteasome is a complex of ATP-dependent proteases found in the cytoplasm and the nucleus [16]. Autophagy is a distinct degradation pathway which transports a set of highly heterogeneous cargo to the vacuole in specific vesicles, called autophagosomes [17]. In plants, they are fused with the vacuole, where cargo becomes degraded and its primary molecules are recycled [18]. While general autophagy randomly engulfs parts of the cytosol, selective autophagy uses special cargo adaptors in response to various intra- or extra- cellular signals, to enrich the forming autophagosomes for a certain type of cargo [19,20,21]. Selective autophagy aims at a broad range of cargo, including long-lived proteins, protein complexes, organelles, and protein aggregates. The analysis of selective autophagy in plants is greatly facilitated by the functional and structural conservation of autophagy-related (ATG) proteins that were initially identified in yeast [22,23,24]. About 40 homologs of 18 yeast genes involved in the core autophagy machinery have been identified in *Arabidopsis thaliana* [19,20,21,25]. All autophagy-deficient plants exhibit hypersensitivity to carbon and nitrogen starvation, pointing to a central role for autophagy in nutrient recycling [26,27]. The upregulation of *ATG* genes during leaf senescence in Arabidopsis suggests a role for autophagy in nutrient recycling at the end of plant life [21,26,28,29,30]. Arabidopsis contains nine highly-conserved ATG8 proteins that, after processing, coat the autophagosomal membranes and serve as a docking platform for autophagy receptors that selectively recognize and bind the cargo designated for degradation [29,31]. Well-known examples of selective autophagy cargo receptors in mammals include p62 (also named SQSTM1 or sequestosome-1, A170, or ZIP) and NBR1 (neighbor of *BRCA1* gene 1), which are both primarily involved in protein aggregate degradation [32,33,34,35]. The NBR1-like selective autophagy cargo receptors exist in plants as well [36,37], but not in yeast. The tobacco Joka2 and Arabidopsis NBR1 proteins are larger than their animal p62 or NBR1 counterparts, but they share a common domain structure, including the UBA domain at the C-terminus, which enable them to bind ubiquitinated proteins. The exact cargo for plant NBR1-like proteins is unknown, and their selectivity may be mediated by ubiquitin recognition and not by specific protein substrates, as is the case in mammals.

Since most of the metabolically-active iron is bound to sulfur in Fe–S clusters, the coordination between metabolisms of the two nutrients is strongly suggested [5,38]. There is physiological and molecular evidence for such crosstalk in different plant species, which additionally suggests that it seems to be species specific [38]. Grasses (Strategy II plants) use the chelating strategy for iron uptake requiring the synthesis of phytosiderophores [39]. Phytosiderophores are derived from nicotianamine synthesized from three S-adenosyl-methionine molecules; thus, there is a need for a well-balanced sulfur metabolism. Iron deficiency in wheat causes the induction of most of the genes of the sulfur assimilatory pathway despite sufficient sulfur supply, suggesting the connection between sulfur and iron metabolism and the necessity of upregulation of sulfur assimilation to increase the synthesis of phytosiderophores [40,41]. Similarly, under sulfur deficiency, the release of phytosiderophores was reduced; however, when barley plants were resupplied with sulfate, the release of phytosiderophores was enhanced [42]. In dicots, sulfur deficiency conditions render plants unable to fully induce their iron uptake machinery, while under iron limitation, the sulfite reduction is stopped [6,7]. Transcriptomic analyses of 5-week iron starved Arabidopsis roots indicated a downregulation of genes of sulfate assimilation [43]. Also, the vacuolar sulfate exporters were induced in leaves, which was interpreted as a necessity of rebalancing the sulfur metabolism under these conditions [44]. Zuchi et al. (2009) [45] showed that in tomato plants exposed to both sulfur and iron starvation, there is reduced activity of iron transporters, which suggests that sulfur deficiency prevents the typical responses to iron deficiency. However, it was also recently shown that iron limitation strongly reduced total sulfur content in both shoots and roots of tomato plants, leading to an increased transcription of sulfate transporters [6]. Altogether, these findings point to coregulation between the two pathways as one nutrient limitation affects the other’s uptake. Nonetheless, these results are based mostly on the changes in gene expression representing only one side of the coin. In both iron and sulfur metabolisms, there are many posttranscriptional regulatory mechanisms that can modulate the nutrient deficiency responses. In this review, we gathered examples of proteins involved in either iron or sulfur metabolism that are the targets of selective degradation. Those proteins have to be removed after specific intra- or extra- cellular cues to reprogram plant metabolism and sustain homeostasis. Although there are ample examples of such proteins for iron metabolism, data for sulfur metabolism is rather scarce.

## 2. UPS in the Regulation of Iron Homeostasis

**Figure 3:**
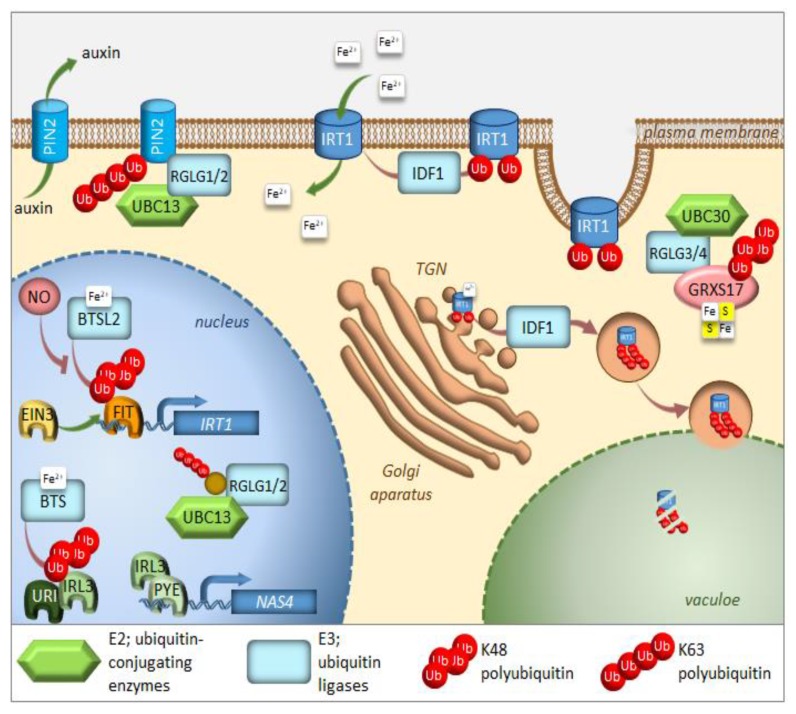
Regulation of iron homeostasis through selective protein degradation. During iron deficiency, FIT induces transcription of the IRT1 iron plasma membrane transporter. EIN3 interacts with FIT and stabilizes its function. IRL3 interacts with PYE to induce transcription of iron-deficiency induced genes, such as *NAS4*. RGLG1/2 ubiquitin ligases are kept within the nucleus, where they interact with UBC13 and target an unknown protein. Under iron-sufficient conditions, after sensing iron availability, two E3 ligases, BTS, and BTSL2 target IRL3 and FIT, respectively, for proteasomal degradation. Through heterodimerization with IRL3, the URI transcription factor responsible for the induction of many genes under iron deficiency, also undergoes proteasomal degradation. IDF1 monoubiquitinates IRT1 at the plasma membrane, causing its endocytosis and eventual targeting to the trans-Golgi network (TGN). There, IRT1, which is able to sense the intracellular iron availability, might be sent to the vacuole for degradation after K63 polyubiqutination via IDF1. RGLG1/2 ubiquitin ligases move from the nucleus to the cytoplasm of root cells, where they target the PIN2 auxin carrier for degradation, thus preventing branching of root hairs. RGLG3/4 ubiquitin ligases together with UBC30 cause K48 polyubiquitination of GRXS17, which takes part in the assembly of Fe–S clusters. Further details and full gene names can be found in the main text.

Iron is an essential nutrient, facilitating photosynthesis, chlorophyll biosynthesis, and a variety of redox reactions. Conversely, homeostasis must be tightly controlled, between uptake, use, and storage of this nutrient, because iron excess inside living cells can be dangerous due to its redox properties. Iron is often present in soils as precipitates that are not readily available for plants. To solubilize iron, plants have evolved various mechanisms to increase iron availability. Arabidopsis responds to iron-limiting conditions by upregulating the expression of basic helix-loop-helix (bHLH) transcription factors, such as Fer-like Iron Deficiency-Induced Transcription Factor (FIT), Popeye (PYE), and PYE homolog IAA-Leu Resistant-3 (ILR3). These transcription factors induce the expression of genes that are needed to increase iron availability and sustain iron homeostasis [46,47,48,49,50]. PYE interacts with additional PYE-like proteins induced during iron-limiting conditions, including bHLH104, bHLH115, and ILR3 (bHLH105) [49,51]. PYE-like proteins are controlled by BRUTUS (BTS), which is an iron-binding E3 ligase found in green algae and plants, and is also induced under iron-limiting conditions [52,53,54] (Figure 3). Ubiquitination activity, together with susceptibility to proteasomal degradation, was proven for BTS [52]. In contrast to *pye-1*, a *BTS* partial loss of function mutation rendered plants more tolerant to iron deficiency, suggesting that BTS is a negative regulator of the iron deficiency response, and that it targets PYE-like proteins for degradation, thus damping the original response to iron deficiency. It should also be noted that the complete loss of function of BTS is lethal under normal growing conditions, which suggests that function of BTS is not limited to iron starvation [55]. Surprisingly, BTS does not interact with PYE directly, but instead, interacts with ILR3, a potential PYE dimerizing partner (Figure 3). During iron-deficient stress, BTS can influence the stability of ILR3, and consequently, indirectly influence PYE activity [49]. BTS is fascinating in its structure. In addition to being an E3 ligase, it also contains three hemerythrin (HHE) domains that can bind iron [53]. This domain is suggested to be critical for sensing whether the iron level is sufficiently restored after the induction of iron uptake machinery [54]. Once activated, BTS targets the positive regulators of iron deficiency response in order to avoid iron overload. Recently, an additional allele of BTS (*bts-3*) was characterized [56]. *Bts-3* mutant overaccumulates iron and other transition elements, in roots, leaves, and seeds, due to constitutive activation of the iron regulon in roots, thus validating the role of BTS as a negative regulator of the response to iron deficiency. However, the constitutive activation of the iron regulon in *bts-3* adds up to only one-fifth of the proper wild type response to iron limitation; more significantly, *bts-3* was unable to activate the iron regulon under prolonged iron depletion [56]. These data suggest that there may be unidentified negative regulator(s), on top of the BTS network, controlling plant responses when iron becomes scarce. Similar regulation can be observed in rice, where the BTS ortholog is called Hemerythrin motif-containing Really interesting new gene and Zinc-finger protein 1 (OsHRZ1) [52]. OsHRZ1 is thought to negatively regulate the iron limitation response by controlling the activity of OsIRO3 (the rice PYE orthologue), which, in turn, coordinates the expression of the bHLH transcription factor gene, *OsIRO2,* and the synthesis of nicotianamine. Very recently, another factor from the bHLH family, OsbHLH058, was found to interact with OsHRZ1 and OsHRZ2; however, this interaction was not due to ubiquitination [57]. The phenotypes of overexpressors and knock-out lines proved that OsbHLH058 positively regulates major iron deficiency responses.

The transcription factor FIT constitutes the most upstream factor in the iron-deficiency signaling pathway. It directly induces the expression of *Ferric Reduction Oxidase 2* (*FRO2*) and *Iron-Regulated Transporter 1* (*IRT1*), the root iron uptake machinery genes [46]. The stability of the FIT protein appeared to be regulated in an iron-dependent manner, with iron deficiency triggering a roughly fivefold decrease in FIT stability [58]. Furthermore, it was proven that the FIT protein is degraded through UPS; however, the E3 ligase specifically recognizing FIT for ubiquitination is still missing. Very recently, FIT was shown to be a ubiquitination target of BRUTUS-like 2 (BTSL2) by an in vitro ubiquitination assay; however, this assay lacked proper controls [54] (Figure 3). The ubiquitination and degradation of FIT are thought necessary to maintain the activity of this regulator due to scavenging of the poorly functional older molecules of this protein from its target promoters [58]. A model was proposed where BTSL, predominantly expressed in the root epidermis and cortex, acts as a primary defense mechanism against excess iron uptake in the roots. The BTS expressed in the stele and leaf tissues, behind the Casparian strip, regulates a second defense mechanism against iron overload by targeting IRL3 [54]. Interestingly, nitric oxide was found as a signal that promoted not only the activation of FIT, but also its stabilization by counteracting proteasomal degradation [59]. Additionally, a link between the iron-deficiency stress response and ethylene signaling has shown UPS involvement in controlling FIT protein levels [50]. Inhibition of ethylene biosynthesis by aminoethoxyvinylglycin (AVG), during the treatment of iron deficiency stress, prevents FIT accumulation, indicating that ethylene signaling is required for stabilization of the FIT protein [50]. FIT interacts with the ethylene signaling transcription factors, Ethylene Insensitive 3 (EIN3) and EIN3-Like1 (EIL1) (Figure 3). This interaction has been shown to reduce FIT degradation and to maintain FIT in an active state, therefore, promoting iron uptake [50]. The loss of function of EIN3 and EIL1 led to a significant reduction of FIT accumulation, even under iron deficiency conditions. Ethylene stimulates *FIT* transcription, which can partly be caused by FIT itself because it is needed to induce its own gene [50,60,61]. Mediator, a large protein complex coordinating the transcription process, has been identified to be involved in the regulation of gene expression during iron deficiency [62]. One of the mediator subunits, MED16, interacts with FIT and recruits it to its promoters to enhance the expression of FIT-dependent genes. Additionally, MED16 was shown to associate with EIN3/EIL1 proteins through subunit MED25, which may play some roles in FIT stabilization [63]. Among other genes, FIT regulates the expression of transcription factor *MYB72,* which has been implicated in iron redistribution through the regulation of nicotianamine synthase 4 (*NAS4*) [58,64]. More recently, MYB72 has been shown to control the synthesis of another type of iron-chelating molecules, known as coumarins, more specifically scopoletin [65]. Interestingly, MYB72 was also found to interact physically with Sulfur LIMitation 1 (SLIM1) [66], one of the very few transcription factors known to regulate sulfur metabolism. How the interaction between MYB72 and SLIM1 impacts iron and/or sulfur homeostasis is currently unknown; however, one can envision a similar mechanism to the interaction observed in EIN3 and FIT. SLIM1 belongs to the same protein family as EIN3 and its degradation through proteasomes has recently been suggested [67].

A short time ago, another piece was added to the iron deficiency response puzzle [68]. Upstream Regulator of IRT1 (URI), basic helix-loop-helix transcription factor, appears to have a central role as an iron-dependent switch. When plants become iron deficient, a phosphorylated form of URI accumulates and induces numerous iron deficiency-induced genes. Under iron resupply, the phosphorylated URI undergoes proteasomal degradation due to heterodimer formation with PYE-like proteins, whose degradation is controlled by E3 ligase BTS. This feedback regulation prevents the overaccumulation of iron and maintains iron homeostasis. As the main iron importer and due to its affinity for other divalent metals, IRT1 is subjected to tight regulation at the transcriptional and posttranscriptional level [69,70,71] (Figure 3). It was surprising to find that IRT1, as a transmembrane protein, localizes predominantly to intracellular compartments, accumulating mainly at the trans-Golgi network (TGN) [72,73]. This demonstrates the importance of intracellular trafficking for iron uptake. In roots, the large cytosolic histidine-rich loop in IRT1 can detect the level of transition elements available for uptake, including iron [74]. Under iron sufficiency, IRT1 employs the machinery to trigger its monoubiquitination, followed by removal from the plasma membrane to be sent back to the TGN [72]. Variants of IRT1, where the ubiquitinatable lysine residues (K154 and K179) were mutated to arginines, caused stabilization of IRT1 and its overaccumulation at the plasma membrane, leading to uncontrolled metal uptake and, subsequently, to plant death [72,75]. The RING-type E3 ubiquitin ligase IRT1 Degradation Factor 1 (IDF1) ubiquitinates IRT1 and, as a result, is depleted from the plasma membrane [76] (Figure 3). Once internalized at the TGN, IRT1 is either reused and sent back to the plasma membrane or targeted for degradation. Interestingly, antibodies against IRT1, demonstrated on immunoblots, that four attached ubiquitin moieties exist in the predominant ubiquitinated form [76]. This suggests that monoubiquitination of K154 and K179 is the signal of IRT1 internalization and that at least two additional lysines might be marked by ubiquitin for further processing in the endomembrane system. The existence of pools of IRT1 decorated with different numbers of monoubiquitination residues may also represent a hierarchy in ubiquitinated residues. Ubiquitin-binding proteins may selectively recognize different monoubiquitinated regions of IRT1, thus controlling the destination of IRT1 along the endocytic pathway. Very recently, it was uncovered that IRT1 acts as a transporter and receptor (transceptor) and is able to directly sense the excess of non-iron metal substrates in the cytoplasm, and subsequently regulates its own degradation [77]. Direct metal binding to a histidine-rich stretch in IRT1 triggers its phosphorylation by the CIPK23 kinase. This facilitates the subsequent recruitment of the IDF1 for K63 polyubiquitination (Figure 3). Non-iron metal excess not only increased the pool of ubiquitinated IRT1 in the cell, but also resulted in a qualitative shift in the type of ubiquitination. Immunoblot analyses revealed that IRT1 is decorated with K63-linked ubiquitin chains that have previously been associated with endocytosis and autophagy [12,13,78]. The authors clearly showed that K63 polyubiquitinated IRT1 is trafficked to the vacuole (Figure 3). This feedback-mediated autoregulation of IRT1 represents a form of iron sensing and is definitely the first line of defense in avoiding an excess of iron or other IRT1 substrates like manganese, zinc, cobalt, and the non-essential element cadmium.

The E2 ubiquitin conjugase UBC13, the only E2 protein that is capable of catalyzing the formation of non-canonical K63-linked ubiquitin chains, has recently been linked to the response of Arabidopsis to iron deficiency. When adapting to reduced iron availability, Arabidopsis develops root hairs that are branched at the base [79]. Mutations in *UBC13A* abolished the branched root hair phenotype. Interestingly, mutations in *RING Domain Ligase 1/2* genes (*RGLG1/2*), encoding UBC13 interacting E3 ubiquitin ligases, caused constitutive root hair branching [80]. A model for the function of K63-linked ubiquitination in root hair cells was proposed [81] (Figure 3). Under iron-sufficient conditions, RGLG1/2 targets a protein that acts as an inhibitor of proper root hair initiation, putatively via an auxin responsiveness reduction. This protein may act directly or indirectly on auxin distribution. In iron-deficient roots, RGLG1/2 moves from the plasma membrane to the nucleus, where it interacts with UBC13, thus releasing its targeted protein [82]. This may result in a decreased auxin concentration. The authors speculate further that that protein could be PIN-Formed 2 (PIN2), a plasma membrane-localized auxin carrier protein. RGLG proteins were actually shown to be involved in the proteolytic turnover of PIN2 via K63-linked ubiquitination [83] (Figure 3). The loss of PIN2 ubiquitination interferes with vacuolar targeting, stabilizes PIN2, decreases auxin levels in roots and, ultimately, leads to the branching of root hairs.

Other members of the RGLG family, RGLG3/4, were recently shown to target monothiol glutaredoxin, GRXS17, for proteasomal degradation [84]. The authors identified the cognate E2 enzyme as UBC30, which is closely related to human HsUbcH5b and, therefore, is probably involved in K48 polyubiquitination (Figure 3). GRXs are present in most organisms and their main function is to control the redox state of proteins. GRXS17 was shown to be necessary for temperature stress and auxin perception [85]. Although none of these functions directly link GRXS17 with iron homeostasis, multidomain GRXs are involved in iron signaling and distribution in yeast. Grx3 and Grx4, the yeast orthologs of GRXS17, can inhibit iron-responsive gene expression, by complexing with the iron-responsive transcription factor activator of ferrous transport 1, RCS1 [86]. The domain architecture of Arabidopsis GRXS17 resembles that of its closest human ortholog HsPICOT, which is involved in the regulation of iron homeostasis, as well as assembly and trafficking of iron-sulfur (Fe–S) clusters [87]. In another paper from this group, Inigo et al. proved that GRXS17 associates with most known cytosolic Fe–S assembly components, putatively to function as a [2Fe-2S] specific adaptor for this complex [88].

The COP9 signalosome (CSN) is an eight-subunit complex that regulates the activity of CULLIN-RING E3 ubiquitin ligases (CRLs) [89]. Recently, CSN has been proven to regulate the proteasome-mediated degradation of iron deficiency-inducible transcription factor, IDEF1, in rice [90]. The decrease in CSN activity followed the accumulation of IDEF1 in the early stages of iron deficiency. Bioinformatic analysis of existing microarray datasets for a set of iron-deficiency-responsive genes was conducted against the transcriptome of Arabidopsis mutants, which are lacking a functional CSN [91]. Hundreds of iron-deficiency-responsive genes appeared to be misregulated, underlying the fact that the correct transcriptional response to iron deficiency requires an intact COP9 signalosome in Arabidopsis and a properly functioning UPS.

In apple (*Malus domestica*), a bHLH transcription factor, *MdbHLH104,* has been found to function in iron acquisition by inducing the transcription of *MdAHA8*, thus modulating the activity of plasma membrane H^+^-ATPases that mediate rhizosphere acidification and iron uptake under iron-deficient conditions [92]. Two MdbHLH104-interacting BTB-TAZ proteins, MdBT1 and MdBT2, were next identified by screening with a yeast two-hybrid method [92]. Subsequently, their role in the ubiquitin-mediated degradation of the MdbHLH104 protein was proved, especially under conditions of iron surplus. MdBT proteins, that accumulate under oversupply of iron, interact with cullin MdCUL3 to bridge the formation of the E3 ligase complex and negatively regulate iron uptake. Conversely, MdbHLH104 is a direct target of the SUMO E3 ligase, MdSIZ1 [93]. Sumolyation, in most cases, serves to antagonize the effects of ubiquitination on target proteins [94]. MdbHLH104 was sumoylated at positions K139 and K153, especially under conditions of iron deficiency, and this modification was required for MdbHLH104 protein stability. Moreover, sumolyation seems to be superior to ubiquitination because MdSIZ1-mediated sumoylation of MdbHLH104 inhibited its ubiquitination. The sumoylation of MdbHLH104 promoted the expression of *MdbHLH38* and *MdbHLH39* and activated the *MdFRO2* and *MdIRT1* genes encoding iron transporters. Therefore, stabilized MdbHLH104 enhances H^+^-ATPase-mediated rhizosphere acidification, but also plays a role in the reduction and absorption of iron to improve tolerance toward iron deficiency in apple plants. The ubiquitination/sumolyation regulatory module of MdbHLH104 serves to sustain iron homeostasis.

## 3. Autophagy in the Regulation of Iron Homeostasis

Iron remobilization is impaired in vegetative tissues of autophagy-deficient plants. Focusing on ATG5, which is essential for autophagosome formation [95], the authors show that autophagy-dependent remobilization is critical for optimal translocation of zinc, manganese, and iron from vegetative organs to seeds. The mechanisms involved in iron remobilization from senescent source organs during seed formation are not well characterized. During senescence, numerous genes involved in degradation mechanisms, including genes involved in autophagy, are upregulated following a well-established schedule [30]. An *atg5* mutant exhibits robust increases in metal concentrations of its dry mass remaining after the completion of its life cycle, confirming that remobilization of metal micronutrients to seeds is impaired [96]. Indeed, the *atg5* mutant showed a decrease in seed iron concentration and a drastic reduction in iron translocation efficiency to seeds. Interestingly, this defect could be alleviated by providing iron to the roots only when *atg5* premature senescence was prevented by an additional mutation in the salicylic acid biosynthesis pathway. Together with data from the ^57^Fe pulse labeling experiment, it suggests that iron taken up by the roots during seed formation contributes indirectly to seed iron content. The authors clearly proved that autophagy is a crucial component of micronutrient filling in seeds, and thus, is a strong determinant of seed quality [96].

Interestingly, the link between autophagy and iron metabolism was also suggested by characterization of the AtNEET protein in Arabidopsis. NEET proteins were found to suppress the activation of autophagy and apoptosis in cancer cells, and as iron-sulfur cluster proteins coordinate iron metabolism and Reactive Oxygen Species (ROS) homeostasis in mammalian cells [97]. Also in Arabidopsis, AtNEET, the sole member of the NEET protein family, was proposed to play similar roles [98]. So far there are no reports on AtNEET involvement in autophagy; however, knock-down mutants and RNAi lines with suppressed expression of AtNEET displayed early senescence and accumulated higher levels of iron and ROS. The lack of true knock-out mutants for AtNEET suggests that it is an essential gene. To advance the study of AtNEET function in plants, a study was undertaken using the dominant-negative strategy to disrupt its function by overexpressing the highly stable H89C variant of AtNEET [99]. The deprivation of AtNEET triggers leaf-associated iron-deficiency responses, elevated iron content in chloroplasts, chlorosis, structural damage to chloroplasts, and high seedling mortality. In mammals, a NEET family protein, Nutrient-deprivation Autophagy Factor-1 (NAF-1) interacts with BCL-2 to antagonize Beclin 1, which stimulates the formation of autophagosomes [100]. The orthologues of BCL-2 and Beclin 1, namely VPS15 and ATG6 respectively, are present in Arabidopsis; however, they are poorly characterized [101].

Recent research highlights an iron- and ROS-dependent cell death phenomenon called ferroptosis in plants. The main hallmarks of ferroptosis are cytoplasmic retraction, shrunken mitochondria, and the formation of lytic vacuoles [102]. At the molecular level, ROS, such as H_2_O_2_, produced by the action of plasma membrane NADPH oxidases, and iron are needed for ferroptotic cell death. Other conditions include the availability of appropriate lipid substrates, such as polyunsaturated fatty acids that may undergo lipid peroxidation, and the lack of antioxidant glutathione that suppresses lipid peroxidation. Highly toxic hydroxyl radicals are generated when ROS react with lipids in the presence of iron. Cysteine is required for glutathione biosynthesis and its reduced availability due to sulfur deprivation conditions, leads to glutathione depletion and lowers the cell’s potential for removal of toxic radicals [102]. Ferroptosis plays an essential role during biotic and abiotic stress adaptation in plants [103,104]. The exposure of Arabidopsis root hair cells to heat shock triggers cell death with the hallmarks of ferroptosis and is accompanied by elevated iron and ROS, but reduced glutathione levels [103]. Although mostly distinct, ferroptotic cell death triggered by heat shock seems to share certain commonalities with other types of cell death (eg. caspases, a class of cysteine-proteases involved in apoptosis). A potential way to improve heat stress tolerance in plants could be to avoid ferroptotic cell death by chemical or genetic means, as presented by Distefano et al. Nevertheless, it is unclear why this disadvantageous cell death was preserved through evolution. It is evident that the antioxidant machinery can no longer neutralize the excessive accumulation of toxic radicals when stress is strong, such as during heat shock, and when a certain threshold is reached this may lead to plant cell death. Another recent study [104] found that the HR-like cell death in rice epidermal cells caused by the avirulent fungal pathogen, *Magnaporthe oryzae,* that causes blast disease is another example of ferroptosis. Elevated levels of iron and ROS accompanied *M. oryzae*-mediated cell death and that phenomenom could be inhibited by iron chelators and Ferrostatin 1. In contrast, erastin, a small molecule that causes glutathione depletion triggering iron-dependent ROS accumulation, promoted virulent *M. oryzae*-induced ferroptotic cell death. It is interesting to note that iron accumulation is not observed during rice infection by a virulent *M. oryzae* isolate. This suggests that virulent *M. oryzae* isolates are able to interfere with ferroptosis during plant infection. Indeed, AVR-Pii, an effector from this pathogen, is known to suppress ROS accumulation during plant infection by inhibiting the rice NADP-malic enzyme [105]. This study [104] is therefore the first case to show the beneficial role of ferroptotic cell death. Although ferroptosis is a relatively new concept in plants, the re-examination of many controlled cell death events reported previously might reveal proof for actual ferroptotic cell death. Recently, a link between ferroptosis and selective autophagy was proven in human cancer cells, where high levels of autophagy have been associated with ferroptosis [106]. p62, a homolog of plant selective autophagy cargo receptor NBR1, degrades the core circadian clock protein ARNTL, thus facilitating ferroptosis induction. Additionally, Nuclear receptor COactivator 4 (NCOA4) is a selective cargo receptor in mammals that mediates the autophagic degradation of ferritin (“ferritinophagy”), the main iron storage complex [107]. Iron released to the cytosol from ferritin is necessary to generate ROS; thus, ferroptosis sensitivity has recently been shown to be modulated by ferritinophagy [108]. So far the role of autophagy in the degradation of ferritin has not been demonstrated in plants.

## 4. UPS in the Regulation of Sulfur Homeostasis

The examples of specific proteins involved in sulfur metabolism and that are substrates of UPS are really scarce. Recently, SLIM1, the only identified transcription factor specifically regulating the genes during sulfur deficiency, was shown to undergo proteasomal degradation [67]. SLIM1 belongs to a small plant-specific multigenic family, of which several members were cloned and described in distinct species [109]. It was demonstrated that SLIM1 can interact with EIN3 Binding F-box 1 (EBF1), which is an E3 ligase described to regulate the abundance of the close SLIM1 homolog, EIN3 [67,110]. It was not determined whether this interaction is needed for ubiquitination and subsequent proteasomal degradation. The authors also proved that proteasomal degradation is essential for proper transcriptomic response to sulfur deficiency. The genes connected with sulfur metabolism were regulated to a lesser extent in the *rpt2a* mutant, which has a proteasomal malfunction due to the mutation in one of the 26S proteasome regulatory subunits, while genes encoding tRNAs and snoRNAs were highly upregulated. This phenomenon was explained as a potential upregulation of ribosome biogenesis or the posttranscriptional rescue mechanism generating very small RNA structures that are able to reduce the level of transcripts encoding proteins which cannot be effectively degraded. Additionally, several genes encoding E3 ligases were identified to be specifically regulated by sulfur deficiency [67].

**Figure 4:**
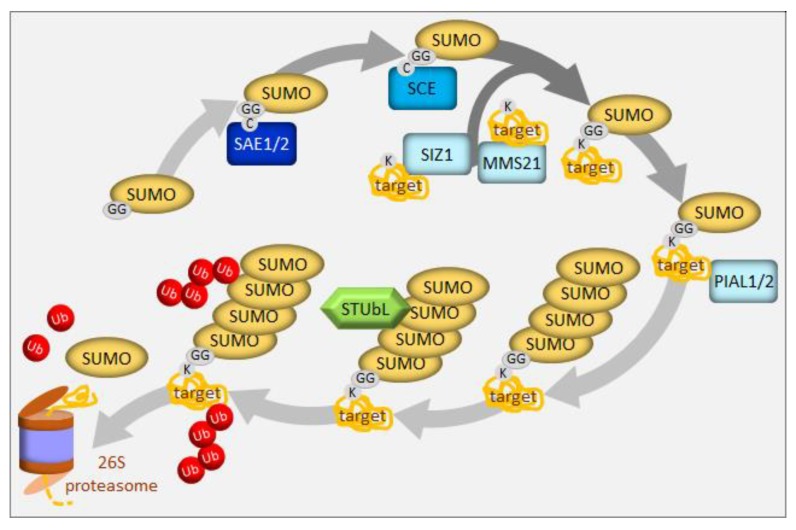
The SUMO conjugation system. The SAE (El enzyme) with its two subunits (SAE1 and SAE2) forms a thioester bond with the reactive carboxyl group of the C-terminal glycine of SUMO to prepare for its transfer to the SCE (E2 enzyme). SCE transfers SUMO to a target protein with the aid of a third enzyme (E3), the SUMO ligase SIZ1 or MMS21. Proteins modified by SUMO may undergo regulated proteolysis started by the formation of a polySUMO chain through the activity of SUMO ligases (E4), such as PIAL1/2. This chain recruits STUbLs, which attach polyubiquitin chains (Ub) both to SUMO and the target protein, thus marking it for degradation by the 26S proteasome.

Sumoylation, the conjugation of the small ubiquitin-related modifier (SUMO) to substrate proteins, is an essential posttranslational modification in plants (for the latest review see: [111]). SUMO is activated by SUMO activating enzyme (SAE) and transferred to a SUMO conjugating enzyme (SCE) (Figure 4). Either alone or with the help of E3 ligases, SCE links the SUMO carboxyl terminus to a lysine residue in the substrate protein. Substrates typically carry a single SUMO moiety. In contrast to the analogous ubiquitination process, which uses a large variety of E3 ligases to target particular substrates, sumoylation employs a surprisingly small number of components, contrasting with its wide spectrum of substrates. In particular, SCE, the product of an essential single-copy gene in Arabidopsis [112], can directly transfer SUMO to a significant fraction of substrates. Only three E3 ligases of the SUMO pathway have been described in Arabidopsis to date, SAP and MIZ1 (SIZ1), Methyl Methanesulfonate-Sensitive 21/High Ploidy 2 (MMS21/HPY2), and the Protein Inhibitors of Activated STAT-Like 1/2 (PIAL1/2), which all contain the SP(SIZ-PIAS)-RING domain [111] (Figure 4). Mutant analysis suggests that PIAL1 and 2 are involved in sulfate assimilation and metabolism [113]. The double *pial1pial2* mutant showed higher sulfate, cysteine, and glutathione levels than the wild type, as well as diminished transcript levels of key genes for sulfur transport and metabolism. In contrast, methionine was decreased and that together with the unchanged level of homocysteine suggests that these mutants may have a lesser capacity to convert cysteine to homocysteine, and subsequently to methionine. This explanation is reinforced by a tentatively higher threonine content and a significantly higher serine content, a precursor to cysteine. The increased flow of cysteine into glutathione might be an effect of the deregulated methionine formation, usage via the Yang cycle, or the deregulation of the enzyme methionine-γ lyase, degrading methionine [113]. Some of these steps might require PIAL1/2 assistance; however, the specific sumoylation substrates remain unknown. In vitro studies revealed that both function to enhance the formation of SUMO chains, in contrast to SIZ1, which adds only single SUMO molecule to the substrate. The authors propose that SUMO chain formation can lead to the removal of SUMO substrates, which explains why the *pial1pial2* mutant contains more SUMO conjugates under stress conditions. SUMO chains on substrates can create binding sites for a family of SIM-containing SUMO-Targeted Ubiquitin Ligases (STUbLs), and thus, channel them into the UPS (Figure 4). STUbLs have been identified in plants, but await biochemical analyses [114]. Thus, as part of their in vivo activity of extending mono-SUMO into a chain, PIAL proteins can channel SUMO substrates into the major proteolytic pathway of the cell [113].

## 5. Selective Degradation via Autophagy in the Regulation of Sulfur Homeostasis

Very recently, the involvement of a selective autophagy cargo receptor, NBR1, in plant response to a sulfur deficit was reported [115]. It was shown that tobacco and Arabidopsis plants exposed to a sulfur deficit have elevated levels of *NBR1* transcripts, especially in root tissue, which might reflect an increased demand for NBR1 under such conditions [37,115]. The elevated number of autophagosomes was observed in roots of sulfur-starved Arabidopsis, proving the induction of autophagy under such conditions [115]. Moreover, the tobacco homolog of NBR1 was shown to strongly interact with the plant-specific family of LSU proteins (response to Low Sulfur) identified as important stress hubs involved in multiple protein-protein interactions [37,116]. The precise function of these proteins is still unknown; however, they are encoded by the genes strongly and specifically induced by a sulfur deficit [117]. Transcriptome analysis of NBR1 overexpressing plants pointed out differences in gene expression during a sulfur deficit response [115]. Significant over-representation of the cytoplasmic ribosomal gene family was observed in genes upregulated by sulfur deficiency in wild type and NBR1 overexpressor roots. The differences suggest that NBR1 could be involved in ribosome remodeling during a plants’ response to sulfur deficiency. The over-representation of the same family in the set of proteins copurifying with NBR1 supported this conclusion. One of the ribosomal proteins, namely ribosomal protein 6 from a small subunit (RPS6) was further proven to directly interact with NBR1 [115].

The autophagy process is tightly controlled to avoid excessive degradation of cellular content. Under normal conditions, this process runs at a low, baseline level and increases when developmental and/or nutritional signals promote assembly of the ATG1/ATG13 autophagy initiation complex [118]. The formation of this complex is negatively regulated by the Target of Rapamycin (TOR) kinase. TOR is active under nutrient-rich conditions, when it increases cell growth and translation whilst preventing autophagy, but it is inhibited during nutrient deficiency [119]. TOR acts as a master regulator of cellular and developmental processes in response to a variety of environmental and metabolic conditions. In animals, the most potent activators of TOR are amino acids; however, the amino acid-sensing transducers of TOR, established in yeast and humans, do not exist in plants [120]. Dong et al. [121] addressed the relevance of TOR for sensing amino acid cysteine in Arabidopsis. In plants, as photo-autotrophic organisms, cysteine is the metabolic hub that integrates the products of sulfate, nitrate, and CO_2_ assimilation pathways. Interestingly, plants detect the presence of cysteine precursors, rather than cysteine itself [121]. This unique mechanism allows plants to differentiate between carbon/nitrogen limitations, rather than sulfur limitation, for the biosynthesis of amino acids. Limitation of the sulfur precursor is transduced to TOR by downregulation of glucose metabolism, while the carbon/nitrogen precursor availability is sensed by the kinase General Control Non-Derepressible 2 (GCN2). GCN2 is conserved in metazoan animals, fungi, and plants and it is selectively stimulated by amino acid depletion to trigger repression of global protein synthesis, while simultaneously inducing the translation of specific proteins [122]. The differential activation of both sensor kinases regulates translation efficiency and tunes up sulfur uptake, together with the remobilization of internal resources of nutrients by autophagy, to coordinate growth with nutrient limitation.

The effect of autophagy defects on leaf metabolism were determined through large-scale proteomic and lipidomic analyses of *atg5* mutant under different nitrogen and sulfur growth conditions [123]. *Atg5* mutant displays a higher level of proteins in leaf tissue than wild type plants and exhibits overstimulation of peptidases [27]. It appears that in the absence of autophagosomal traffic, proteases and their substrates seemed unable to meet, which led to overaccumulation of non-degraded proteins. This report showed the strong impact of impaired autophagy on ER stress and reveals the role of autophagy in controlling lipid homeostasis and endomembrane composition [123]. Lipidomic analyses revealed changes in the concentrations of sphingolipids, phospholipids, and galactolipids in *atg5* mutant. Decreases in chloroplast proteins, which are mostly involved in photosynthesis, the Calvin cycle, and fatty acids biosynthesis, and galactolipids, under sulfur limiting conditions, indicated that chloroplasts were used as lipid reservoirs for β-oxidation in *atg5* mutant. An increased phospholipid content in *atg5* mutant may reflect the enrichment of ER in phospholipids, which cannot be used for the formation of phagophores. In the sulfur limiting conditions under which autophagy is usually induced, the overabundance of phospholipids in *atg5* mutant was escalated. Surprisingly, proteomic studies showed an increase in the relative abundance of the three catalases in *atg5* mutant that was undetectable under low sulfur conditions. The authors showed that the oxidative stress affecting *atg5* mutant was more severe under control conditions than under nutrient limitation. Although enzymes involved in amino acid catabolism in the cytosol and mitochondria [124] were also more abundant in *atg5* mutant, they were more specifically increased under sulfur limitation conditions. The Gene Ontology search for proteins induced by sulfur limitation in *atg5* mutant was primarily characterized by a significant enrichment in aquaporins. The changes in endomembrane lipid composition in *atg5* may strongly modify nutrient transport and long-distance signaling. Such disorders could explain the defects of autophagy mutants in nutrient allocation ([125] for a review). Although there are no other data on sulfur metabolism and its homeostasis in autophagy mutants, we could assume similar mechanisms. Further studies revealed the role of autophagy in sulfur management at the whole plant level by the application of _34_S isotope labeling on *atg5* mutant and wild type plants [126]. Sulfur remobilization from the rosette to seed was impaired in *atg5* mutant and most of the amino acids, including methionine and cysteine, accumulated in the rosette leaves of *atg5* mutant, irrespective of sulfur supply. The sulfate concentration was evidently higher in the *atg5* mutant than in the wild type plants, which might be related to their lower growth rate, resulting in a lower dilution of sulfur. However, it suggests that sulfate is not efficiently reduced and assimilated when autophagy is impaired.

The cellular function of hydrogen sulfide (H_2_S) in mammals is comparable to that of nitric oxide [127]. The importance of H_2_S homeostasis and its participation in stress signaling and protection are recognized in plants as well [128]. The endogenous production of H_2_S by plant cells is related to the biosynthesis and metabolism of cysteine, with the chloroplast as a major source [129]. Most of the sulfide present inside the chloroplast is dissociated into its ionic form HS-, which cannot freely permeate the membrane and requires an active transporter, which is currently unknown. In the cytosol, H_2_S that is generated from cysteine by different cysteine-degrading enzymes, such as cysteine desulfhydrases (CDES) and cysteine desulfurases (NifS-like) [130]. DES1 is the only L-Cys desulfhydrase present in the Arabidopsis cytosol that catalyzes the desulfuration of L-cysteine to sulfide [131]. Analysis of the Arabidopsis *des1* mutant, which had impaired cytosolic H_2_S production from cysteine, resulted in the conclusion that H_2_S acts as an inhibitor of autophagy induced by nutrient deprivation [132]. Senescence-associated vacuoles and induced autophagy were observed in *des1* mutant and the phenotype could be rescued by the addition of exogenous sulfide. The transcriptomic data confirmed induction of autophagy in the *des1* mutant, in which several *ATG8* and *ATG12* genes were upregulated. Moreover, additional sulfide rescued autophagy activation, resulting from dark-induced carbon starvation in wild type Arabidopsis plants. This led to the conclusion that H_2_S can also serve as a signaling molecule in autophagy. The mechanism of autophagy inhibition by H_2_S is poorly understood. However, it may be based on the hypothesis of Gotor and colleagues [132], that enzymes involved in ubiquitination or any other proteins involved in the initiation or completion of the autophagosome are reversibly modified after translation by S-sulfhydration (also known as persulfidation) at the reactive cysteine residue(s). Such modification usually increases the catalytic activity of the target proteins because it merely changes an -SH to an -SSH, enhancing the enzymes’ chemical reactivity and possibly improving access to their respective targets [133]. However, the significance of S-sulfhydration in autophagy has not been sufficiently explored and proteomic analysis performed on wild type plants revealed the susceptibility of only four autophagy-related proteins, ATG18a, ATG3, ATG5, and ATG7, to persulfidation [134]. Incidentally, the role of H_2_S in modulating iron availability in maize seedlings grown in iron-deficient conditions was recently reported [135]. It completely prevented leaf interveinal chlorosis and increased chlorophyll biosynthesis, chloroplast development, and photosynthesis in plants. H_2_S treatment increased iron accumulation by changing the expression levels of iron homeostasis- and sulfur metabolism-related genes.

## 6. Conclusions

Plant tolerance of adverse growth conditions, including nutrient fluctuations, involves developmental, physiological, and biochemical changes, which reduce damage and restore homeostasis. Adaptation to the changing environment has an effect on growth, development, and yield. Therefore, determination of the mechanisms controlling stress tolerance is crucial. Plant responses to iron and sulfur deficiencies appear to be mediated by both transcriptional regulation and protein quality control via ubiquitination. The discovery of E3 ubiquitin ligases that are involved in abiotic stress responses provides a direct link between the UPS and plant stress tolerance. Only a very small fraction of over 1500 known Arabidopsis E3 ligases have defined roles in abiotic stress tolerance. The lack of substrate identity hinders our understanding of the regulatory role of E3s during plant stress responses. Once a substrate is identified, the mechanism of regulation by ubiquitination can be established. Currently, very little is known about the specific regulation of plant E3 ligases in response to external stimuli, besides the fact that the expression of many E3-encoding genes is stress-regulated. Knowledge of the mechanism of stress signal-mediated up or downregulation of E3 ligase activity will expand our understanding of cellular changes required for adaptation to changing environmental conditions.

The selectivity of protein degradation by UPS is a well-established process. The discovery of the selectivity of plant autophagy by the identification of selective autophagy receptors that can bind specific cargo destined for degradation by ubiquitination sheds new light on the autophagy process. It is still very difficult to clearly distinguish between proteins destined to be degraded in plant cells by either UPS or autophagy, and it will be extremely valuable to establish if unique ubiquitin signatures exist for these processes.

## Abbreviations

ATG	AuTophaGy-related proteins
ROS	Reactive Oxygen Species
SUMO	small ubiquitin-related modifier
TGN	trans-Golgi network
Ub	Ubiquitin
UPS	Ubiquitin-Proteasome System
